# Selecting stable rice mutants with linear mixed models (LMM) and stability indexes

**DOI:** 10.18699/vjgb-26-25

**Published:** 2026-04

**Authors:** P. Sharifi, A.A. Ebadi, M.T. Hallajian, H. Aminpanah

**Affiliations:** Department of Agronomy, Ra. C., Islamic Azad University, Rasht, Iran; Rice Research Institute of Iran, Agricultural Research, Education and Extension Organization (AREEO), Rasht, Iran; Nuclear Science and Technology Research Institute of Iran, Tehran, Iran; Department of Agronomy, Ra. C., Islamic Azad University, Rasht, Iran

**Keywords:** genotype-by-environment interaction, MTSI, mutation breeding, simultaneous selection index, WAASB, взаимодействие генотип–среда, MTSI, мутационная селекция, индекс одновременного отбора, WAASB

## Abstract

Mutation serves as a pivotal source of diversity in plant breeding. This study focused on identifying stable rice mutant lines. Fourteen rice mutant lines, along with four conventional cultivars, were evaluated in a randomized complete block design with three replicates across three Iranian locations (Rasht, ChaparSar, and Fars province) during two growing seasons (2015, 2016). All statistical analyses were performed using the ‘metan’ (multi-environment trial analysis) R package. Single-environment ANOVA indicated significant genotypic effects for all traits. Likelihood ratio tests (LRTs) confirmed significant environment and genotype-by-environment interaction (GEI) effects for all traits. The first three principal components (PCs) captured 68.13, 14.46, and 9.76 % of the GEI variation, respectively. Heatmap visualization of yield performance and WAASB (weighted average of absolute scores based on best linear unbiased prediction, BLUP) highlighted genotypes G3, G9, G6, G12, and G5 as both high-yielding and stable. Multi-trait stability index (MTSI) analysis, designed to reveal genotypic strengths and weaknesses, selected only genotypes G7, G5, and G1. The top five genotypes based on the harmonic mean of the relative performance of genotypic values (HMRPGV) were G5, G12, G7, G2, and G1. The superior performance of certain mutants demonstrates that mutation has effectively generated significant genetic diversity. Notably, genotypes G12, G5, and G9 exhibited a clear advantage over the other genotypes and warrant consideration for selection or cultivar release; however, only G5 was selected based on all traits in the MTSI index and could therefore undergo selection or cultivar introduction processes.

## Abstract (ru)

Мутационные изменения служат основным источником разнообразия в селекции растений. Данное исследование было сосредоточено на выявлении стабильных мутантных линий риса. Четырнадцать мутантных линий и четыре обычных сорта риса были оценены в рандомизированном посеве с тремя повторениями в трех регионах Ирана (Решт, Чапарсар и провинция Фарс) в течение двух вегетационных сезонов 2015 и 2016 гг. Все статистические анализы выполнены с использованием пакета R ‘metan’ (multi-environment trial analysis). Однофакторный ANOVA показал значимые генотипические эффекты для всех признаков. Тесты отношения правдоподобия (LRT) подтвердили значимые эффекты среды и взаимодействия генотип–среда (GEI) для всех признаков. Первые три главные компоненты определяли 68.13, 14.46 и 9.76 % вариаций GEI соответственно. Визуализация тепловой карты для выраженности урожайности и WAASB (взвешенное среднее абсолютных баллов на основе наилучшего линейного несмещенного прогноза, BLUP) позволила выделить генотипы G3, G9, G6, G12 и G5 как высокоурожайные и стабильные. Анализ с помощью индекса мультипризнаковой стабильности (MTSI), разработанный для выявления сильных и слабых сторон генотипов, отобрал только генотипы G7, G5 и G1. С использованием параметра гармонического среднего относительной производительности генотипических значений (HMRPGV) были отмечены пять лучших генотипов – G5, G12, G7, G2 и G1. Высокая выраженность признаков у некоторых мутантов демонстрирует, что мутационные изменения могут эффективно создавать значительное генетическое разнообразие. В частности, генотипы G12, G5 и G9 имели явное преимущество перед другими генотипами и могут быть использованы для последующей селекции или для создания сорта. Однако только генотип G5 был отобран с учетом индекса MTSI на основе всех признаков и, следовательно, может быть использован в дальнейшем селекционном процессе или для создания сорта

## Introduction

Rice stands as a critical cereal crop, serving as a primary food source for over half of the global population. It assumes a vital role in ensuring food and nutritional security, while concurrently contributing to poverty alleviation (Khush, 2005). Crop production is susceptible to abiotic stresses, including drought and salinity, necessitating the evaluation of genotypes across diverse environments. This approach facilitates the identification of adaptable genotypes suitable for broad or specific environmental conditions (Sharifi, 2020).Mutation is a key source of generating variation in plant breeding programs, allowing for the development of novel traits or the improvement of existing ones. This process relies on alterations within the genetic structure, leading to new heritable traits that can spread within plant populations (Shu, 2012). Mutation breeding is often used to develop cultivars that are more tolerant to biotic and abiotic stresses, enhance quality, or alter other plant characteristics (Ebadi et al., 2019). This strategy is a useful complement to existing germplasm and can help in the development of new cultivars. As plant phenotypes are affected by genotype-by-environment interaction (GEI), stability analysis is essential to understand the performance of genotypes across different environments and guide breeders in selecting superior genotypes (Sharifi et al., 2017). Consequently, yield stability and adaptability analyses in multi-environment trials are a critical component of genotype evaluation programs before a new breeding line is released as a commercial variety (Kang, 1988). This demonstrates the plant’s ability to maintain yield potential despite environmental fluctuations (Yan et al., 2000).Stability analysis has become an important part of research and plant breeding. Various statistical models, including univariate and multivariate methods, are used to assess the stability and adaptability of genotypes. The simultaneous selection index named as grain yield in a single non-parametric index (GSI), which is based on the sum of ranks for yield stability and performance, is often used as a criterion for stability analysis (Farshadfar, 2008). Yan (2016) noted that mean performance and stability are not always equally important when evaluating genotypes. Stability is more of a concern when genotypes are tested in only a few environments. However, when genotypes are adequately tested, information about their stability may already be reflected in their mean performance, because highly unstable genotypes – those that perform very well in some environments but poorly in others – will not be among the top performers across all environments. Newer indices for evaluating stability based on linear mixed models (LMMs) include WAASB (weighted average of absolute scores based on best linear unbiased prediction (BLUP)) and WAASBY (WAASB stability index and yield performance, for simultaneous selection based on yield performance and stability) (Olivoto et al., 2019b). This approach combines additive main effects and multiplicative interaction models (AMMI), graphical tools with the predictive precision of BLUP (Olivoto et al., 2019b). The relative importance of mean performance and stability can vary when making selection decisions, which can change how genotypes are ranked (Yan, 2016). This explains why many breeders and variety evaluation organizations base their decisions only on mean performance, yet still achieve significant breeding progress (Yan, 2016). Given the importance of the WAASBY index, it can be used for all stability indices instead of the GSI (Farshadfar, 2008), allowing for the identification of the most superior genotypes.

In addition to high yield, which remains the primary breeding objective, a cultivar must meet minimum requirements for every trait that is significant to growers, processors, and end-users (Yan, 2021). Consequently, when data on multiple traits are available, the multi-trait stability index (MTSI) has several valuable applications for the simultaneous selection of mean performance and stability. Indeed, the greatest challenge in plant breeding is to combine all desirable traits within a single genotype, as key breeding objectives are often negatively correlated due to genetic linkage or pleiotropy (Yan, 2021). These indices are used in the stability analysis of rice (Sharifi et al., 2021), lentil (Karimizadeh et al., 2020), forage (Santos, Marza, 2020), barley (Ahakpaz et al., 2021), and wheat (Verma, Singh, 2020). An alternative analysis for stability studies based on mixed models is the harmonic mean of genotypic values and of the relative performance of genotypic values (HMRPGV), which provides information on stability, adaptability, and yield performance of genotypes in the same unit and scale as the evaluated trait (Resende, 2007). Selecting genotypes with the highest values of the harmonic mean of genotypic values (HMGV), relative performance of genotypic values (RPGV), and HMRPGV allows for simultaneous selection for yield performance and stability. This approach is used to evaluate grain yield stability in rice (Colombari-Filho et al., 2013), wheat (Coan et al., 2018; Verma, Singh, 2020), and corn (Rodovalho et al., 2015).

deviations from regression to identify stable mutants. Dewi and Dwimahyani (2019) evaluated 12 mutant rice genotypes at 16 locations and estimated yield stability using regression lines. Rahayu (2020) used AMMI analysis to differentiate stable mutant rice lines from 25 genotypes. Relatively few studies have used linear mixed models for rice stability analysis. Donoso-Ñanculao et al. (2015) analyzed 10 rice genotypes using BLUP and identified superior genotypes. Balestre et al. (2010) also used BLUP to analyze the stability of rice genotypes and found that phenotypic means had lower predictive potential compared to BLUP.

The objectives of this study were to (1) evaluate GEI in rice genotypes across diverse environments in Iran, and (2) identify stable and high-yielding mutant lines.

## Materials and methods

**Plant material and experimental design**

Fourteen rice mutant lines, along with their parental cultivars (Hashemi, Tarom, and Khazar) and the Gilaneh variety as a control (Table 1), were evaluated in a randomized complete block design (RCBD) with three replications. The experiment was conducted across three geographically distinct research stations in Iran: Rasht, ChaparSar, and Fars province. Data were collected over two years (2015, 2016). In both years, sowing took place in April, and transplanting was carried out in May at the 4–5 leaf stage. Harvesting occurred in August in both years as well.

**Table 1.**
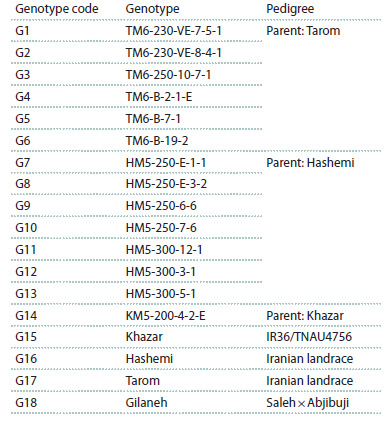
The origin/pedigree of rice genotypes used in this experiment

The rice mutant lines originated from seeds of the parental cultivars subjected to 300 Gray of gamma irradiation from a Cobalt-60 source. Irradiation was performed at the Iranian Agricultural Atomic Research Institute in Tehran in 2009, with seed moisture content maintained at eight percent. Following irradiation, the M1 generation was grown in the field. Surviving plants were self-pollinated, and seeds from the M2 generation were collected. This self-pollination process was repeated through the M5 generation to achieve homozygosity.

Prior to sowing, seeds of all experimental genotypes (mutant lines and cultivars) were surface-sterilized with a 20 % sodium hypochlorite solution for 10 minutes, followed by rinsing with sterile distilled water. This procedure was carried out in May of each year. Seeds were then placed in Petri dishes containing moistened filter paper to initiate germination. Germination occurred in a controlled environment at 35 °C and 70 % humidity. Seedlings were subsequently transplanted into 120 cm×60 cm boxes within greenhouses, ensuring suitable conditions for transplant production

Twenty-five-day-old seedlings (approximately 30 cm tall) were transplanted to the main field at a planting density of 25 plants per square meter. Each plot measured two square meters. Data were collected from ten randomly selected plants within each plot. The following traits were measured: plant height, tiller number, panicle length, number of filled grains per panicle, number of unfilled grains per panicle, fertility percentage, and hundred-grain weight. Grain yield was determined on a per-square-meter basis

**Statistical analysis**

To assess genotypic stability and adaptability, the harmonic mean of genotypic values (HMGV) and the relative performance of genotypic values (RPGV) were calculated. The harmonic mean of the relative performance of genotypic values (HMRPGV) was employed for the simultaneous evaluation of stability, adaptability, and yield. These indices were computed using the following formulas (Resende, 2007):

**Formula. 1-3.**
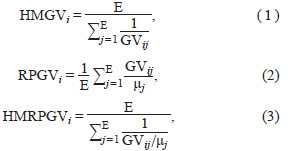
Formula. 1-3.

where μj represents the general mean for the j-th environment; E denotes the number of environments; and GVij : uj+gi+geij indicate the genotypic value of the i-th genotype in the j-th environment. Here, uj is the mean of the j-th environment, while gi and geij are the BLUP values of the i-th genotype and the interaction between the i-th genotype and j-th environment, respectively.

The weighted average of absolute scores from the singular value decomposition (SVD) of BLUP-estimated interaction effects (WAASBi) was computed using equation (4) (Olivoto et al., 2019a):

**Formula. 4.**
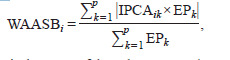
Formula. 4.

where IPCAik is the score of the i-th genotype (or environment) in the k-th IPCA, and EPk is the value of the explained variance by the k-th IPCA. The k-th IPCA is the k-th interaction principal component axis (IPCA) in AMMI, used to compute WAASB. The genotype with the lowest WAASB value is considered the most stable (Olivoto et al., 2019a).

The WAASBY index, which allows simultaneous selection based on grain yield and stability, was derived using the following formula (Olivoto et al., 2019a):

**Formula. 5.**
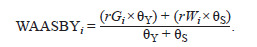
Formula. 5.

Where WAASBYi is the superiority index for the i-th genotype, and θY and θS are the weights for seed yield and stability (WAASB), respectively. The rescaled values (0–100) for the response variable (rGi) and WAASB (rWi) were calculated as follows:

**Formula. 6-7.**
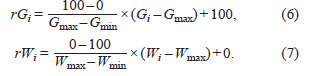
Formula. 6-7.

The multi-trait stability index (MTSI) for the i-th genotype was calculated as follows (Olivoto et al., 2019b):

**Formula. 8.**
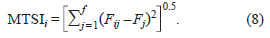
Formula. 8.

Where, Fij is the j-th score of the i-th genotype, and the ideotype (Fj) is an ideal reference genotype with the most desirable values for each trait, f represents the number of traits (variables) included in the analysis. The ideotype has the highest WAASBY (100) for all analyzed variables (Olivoto et al., 2019b). The genotype with the lowest MTSI is then closer to the ideotype and therefore presents a high mean performance and stability (MPE) for all analyzed variables

Selection differential (SD), selection gain (SG), and percentage of selection differential (% SD) represent the difference between the mean of selected genotypes (X) and the entire population, the expected genetic progress, and the relative selection intensity, respectively.

**Formula. 9-11.**
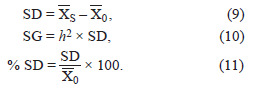
Formula. 9-11.

XS: mean of selected genotypes; X0: mean of the entire population; h2: heritability of the trait.

The selection differential for mean performance and the WAASBY index was calculated for each trait considering a selection intensity of 15 %.

All statistical analyses were performed using the ‘metan’ (multi-environment trial analysis) R package (Olivoto, Lúcio, 2020). An example of statistical analysis using R can be seen in the Supplementary file1.

Supplementary Materials are available in the online version of the paper: https://vavilovj-icg.ru/download/pict-2026-30/appx16.pdf


## Results

**Analysis of variance, estimation of variance components and predicted grain yield**

Single-environment ANOVA indicated significant genotypic effects for plant height, tiller number, panicle length, number of filled grains per panicle, number of unfilled grains per panicle, fertility percentage, hundred-grain weight, and grain yield. Likelihood ratio tests (LRTs) confirmed significant environment and genotype-by-environment interaction (GEI) effects for all traits. Analysis of variance also indicated a significant effect of genotype on all traits (Table 2).

**Table 2.**
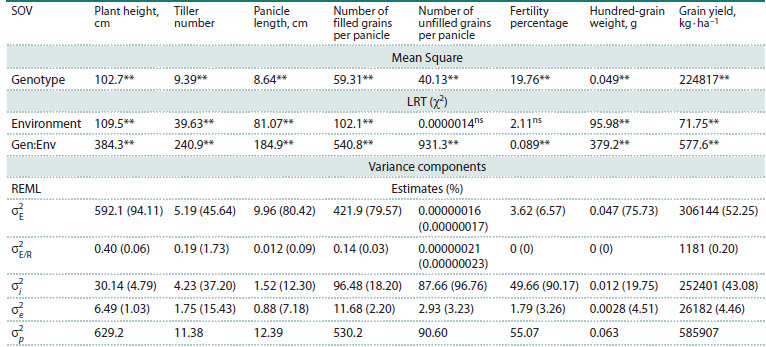
Evaluation of significance of factors for random effects (E and GEI) by LRT (χ2) and for fixed effect (G) by ANOVA and estimation of variance components by REML Note. The numbers in parentheses indicate the percentage of variance accounted for by the phenotypic variance. LRT, likelihood ratio test. σ2 E , environmental variance; σ2 E/R , Block-within-Environment variance (or Environment/Replication variance); σ2 i , Genotype × Environment interaction variance; σ2 e, residual variance; σ2 p, phenotypic variance. ns, non-significant, *, **: significant at 5 % and 1 % probability levels, respectively

**Principal component analysis and biplot interpretation**

**Fig. 1.**
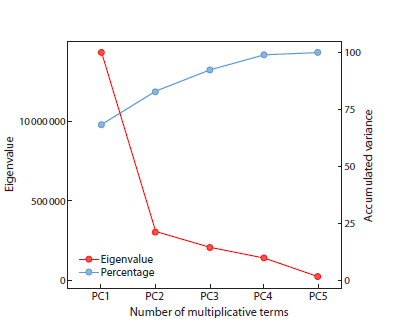
Eigenvalues of the BLUP_GEI matrix for grain yield of 18 rice genotypes across six environments

**Fig. 2.**
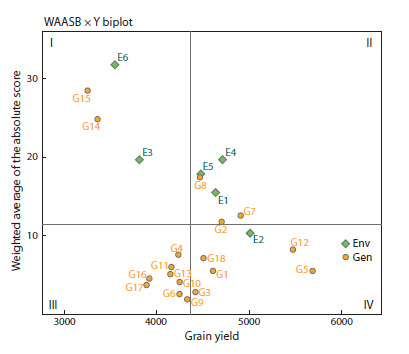
Biplot of grain yield vs WAASB of 18 rice genotypes evaluated over six environments (combinations of two cultivation years in three locations).

**Fig. 3.**
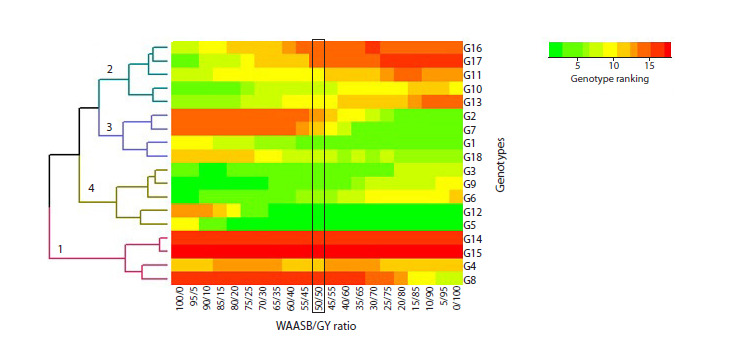
Ranks of 18 rice genotypes with different weights for stability and grain yield.

The first three principal components (PCs) captured 68.13, 14.46, and 9.76 % of the GEI variation, respectively (Fig. 1). A type III biplot (WAASB values vs grain yield, Fig. 2 and Fig. 3) was used to visualize genotypic stability.

Genotypes G14 and G15, along with environments E3 and E6, positioned in the first quadrant, exhibited below-average grain yield and high WAASB values, indicating instability and a substantial contribution to GEI. Conversely, genotypes G2, G7, and G8, and environments E1, E4, and E5, in the second quadrant, showed above-average grain yield but were also unstable with significant GEI contributions. Environments E3, E4, and E6 displayed the highest WAASB values, indicating greater discriminating ability. Genotypes G4, G6, G9–G11, G13, G16, and G17, located in the third quadrant, demonstrated low yield and high stability (low WAASB). The most desirable genotypes – G1, G3, G5, G12, and G18 – occupied the fourth quadrant, characterized by high yield and stability. Environment E2, also in this quadrant, showed high yield performance but low WAASB values, indicating poor genotypic discrimination ability (Fig. 2)

**Genotype ranking based on stability and performance weights**

Figure 3 illustrates genotype rankings based on varying weights assigned to WAASB (stability) and grain yield. Ranking of 18 rice genotypes was based on the WAASBY index, illustrating variations in genotype performance under different weighting schemes for stability (WAASB) versus grain yield (BLUPs). The weights (θ) range from 0 (prioritizing yield alone) to 1 (prioritizing stability alone), with θ = 0.5 representing equal emphasis on both traits. Ranking based solely on stability (WAASB weight = 1) identified genotypes G6, G9, G3, G10, G13, and G17 as most stable. Ranking based solely on yield (yield weight = 1) identified G1, G2, G5, G7, and G12 as the highest-yielding genotypes.

Hierarchical clustering revealed distinct genotype groupings based on stability and yield: (1) G4, G8, G14, and G15 were the least desirable; (2) G10, G11, G13, G16, and G17 were stable but low-yielding; (3) G1, G2, G7, and G18 were high-yielding but unstable; and (4) G3, G5, G6, G9, and G12 (particularly G5) were both high-yielding and stable.

**Factor analysis, multi-trait stability index (MTSI) and genotype selection**

Factor analysis of the WAASBY index retained two principal components (eigenvalue > 1), explaining 71.5 % of the total variation (Table 3). Factorial loadings after varimax rotation and communalities obtained in the factor analysis are represented in Table 4. The eight traits were grouped into two factors: FA1 (agronomic and plant stature traits including plant height (PH), filled grains per panicle (FG), unfilled grains per panicle (UNFG), fertility percentage (FP), hundred-grain weight (HGW), grain yield (GY)), and FA2 (panicle-related traits contain tiller number (TN) and panicle length (PL)). While primarily associated with FA1, FG, UNFG, and GY also exhibited high scores on FA2 (Table 5).

**Table 3.**
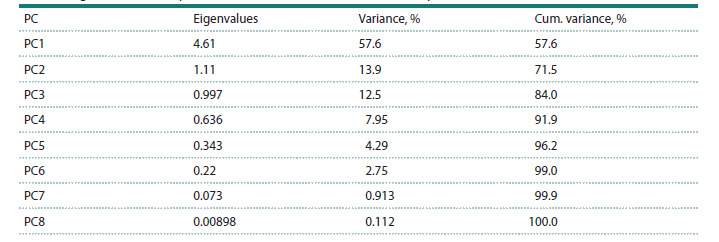
Eigenvalues and explained variance obtained in the factor analysis

**Table 4.**
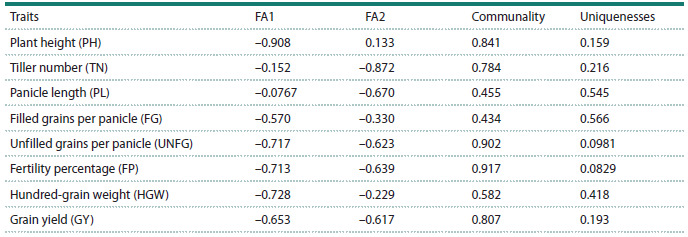
Factorial loadings after varimax rotation, and communalities obtained in the factor analysis

**Table 5.**
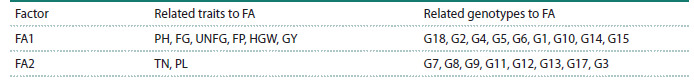
The related traits and genotypes for each factor

**Fig. 4.**
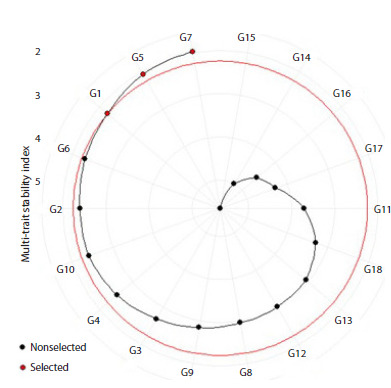
Genotype ranking and genotypes selected for MTSI

Ranking based on MTSI identified G7 (MTSI = 1.97), G5 (MTSI = 2.07), and G1 (MTSI = 2.24) as superior genotypes (Fig. 4, cutoff at MTSI = 2.24). The cutoff value (MTSI = 2.24) was determined following the methodology of Olivoto et al. (2019b), where the Multi-Trait Stability Index (MTSI) ranks genotypes based on their stability and multi-trait performance. This threshold represents the upper limit of the top-performing genotypes (G1, G5, G7), beyond which stability and performance decline significantly. The selection of this cutoff aligns with standard MTSI applications, where natural breaks in the index distribution or percentile-based thresholds are used to distinguish superior genotypes. The genotype with the lowest MTSI is then closer to the ideotype and therefore presents a high MPE for all analyzed variables (Olivoto et al., 2019b).

The MTSI resulted in positive selection differentials (SD) for WAASBY of all traits, ranging from 13 % (TN) to 49.5 % (HGW) (Table 6). Negative SDs for mean performance were observed for PH and UNFG, while positive SDs were noted for the remaining traits. This led to negative selection gains (SG) for PH (–0.35 %) and UNFG (–47.5 %), and positive SG for FG, FP, HGW, GY, TN, and PL (0.996 % ≤ SG ≤ ≤ 16.2 %). These results indicate that the selected genotypes exhibited improved genotypic stability compared to the original population, and indicate that MTSI is effective for genetic breeding.

**Table 6.**
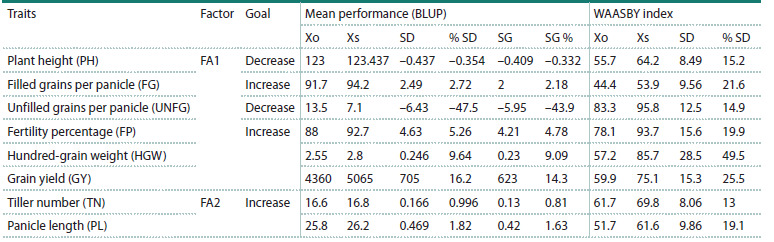
Selection gains for mean performance of 18 rice genotypes obtained in a multi-trait selection with the multi-trait stability index (MTSI) Note. Xo: mean performance (BLUP) and WAASBY index of the original population; Xs: mean performance (BLUP) and WAASBY index of the selected genotypes; SD: selection differential; % SD: percentage of selection differential; SG: selection gain.

**Visualization of strengths and weaknesses**

**Fig. 5.**
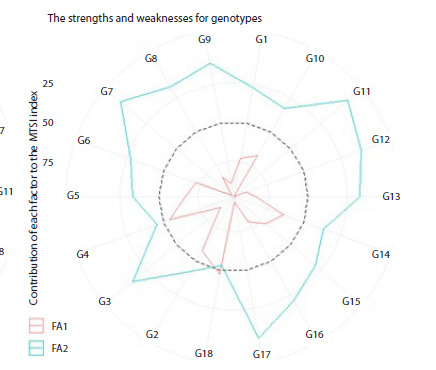
The strengths and weaknesses of genotypes are shown as the proportion of each factor on the computed multi-trait stability index (MTSI) of all genotypes. The smaller the proportion explained by a factor (closer to the external edge), the closer the traits within that factor are to the ideotype. FA1: PH, FG, UNFG, FP, HGW and GY; and FA2: TN and PL

Figure 5 visualizes the strengths and weaknesses of each genotype. The contribution of each factor (FA1 and FA2) to the MTSI is ranked for each genotype, with the most contributing factor near the plot center and the least contributing factor near the plot edge (Olivoto et al., 2019b). Genotypes associated with a specific factor demonstrate high mean performance and stability (low WAASB) for the traits primarily influenced by that factor. Ten genotypes (G1, G2, G4, G5, G6, G10, G14, G15, G16 and G18) showed strengths related to FA1 (Fig. 5), indicating desirable combinations of high mean performance and stability for FA1-related traits: reduced PH and UNFG, and increased FG, FP, HGW, and GY. Eight genotypes (G3, G7, G8, G9, G11, G12, G13, and G17) exhibited strengths related to FA2 (Fig. 5). For G5, the second-ranked genotype by MTSI, significant FA1 contribution confirms its favorable combination of reduced PH and UNFG, and increased FG, FP, HGW, and GY. This genotype exhibited lower plant height and unfilled grains per panicle, and higher tiller number, hundred-grain weight, and grain yield, compared to the overall average. Genotype G12 showed higher tiller number, fertility percentage, and grain yield, along with lower plant height and unfilled grains per panicle. While identified as superior by other indices, G12 did not rank highly using MTSI. This discrepancy is likely due to the low selection gain for traits in FA1, specifically hundred-grain weight.

**Genotypic stability and adaptability assessment using HMRPGV**

Based on the Harmonic Mean of Relative Performance of Genotypic Values (HMRPGV), the top five genotypes for stability and adaptability, relative to check varieties, were G1, G2, G7, G12, and G5. The HMRPGV multiplied by the overall mean (HMRPGV*μ) for these genotypes were 4597, 4680, 4901, 5481, and 5696 kg · ha–1, respectively (Table 7). Selecting these genotypes would result in a 16.35 % increase in grain yield compared to the overall mean (4360 kg · ha–1). This selection, based on stability, adaptability, and yield, highlights the positive response of these genotypes to improved environmental conditions and their consistent performance across diverse environments.

**Table 7.**
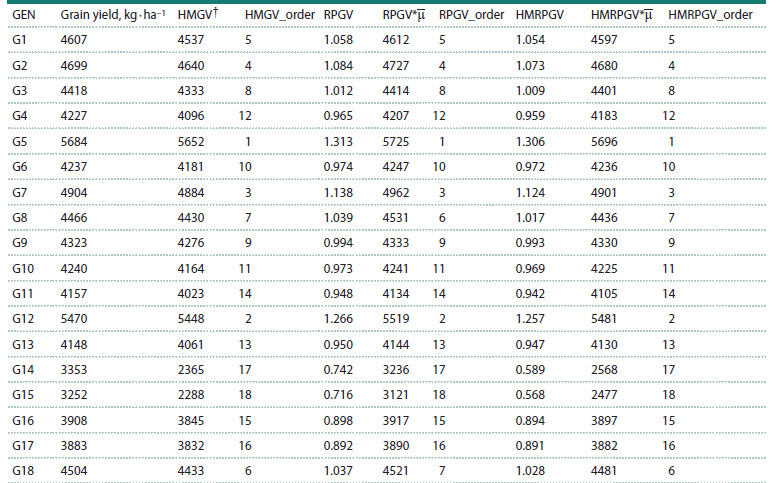
Ranking of the genotypes in all environments evaluated for adaptability parameters of genotypic values for the grain yield of rice genotypes evaluated in six environments Note. † Performance genetic value (RPGV); RPGV*μ: stability of genotypic values (HMGV), adaptability and stability of genotypic values (HMRPGV); HMRPGV*μ: general mean of all environments; the parameters are expressed in kg · ha–1, except for RPGV and HMRPGV.

## Discussion

A broad genetic base is crucial for successful rice breeding programs, and induced mutation represents a valuable technique for expanding genetic diversity (Cheema, 2006). The significant effect of genotype observed across all environments, as indicated by simple analysis of variance, underscores the genetic diversity present within the experimental material. This result suggests that mutation has effectively contributed to a reasonable level of diversity within the genetic material under investigation. This finding aligns with the work of other researchers who have assessed the stability of rice mutant lines and demonstrated a significant impact of mutation in generating diversity with respect to grain yield and other important traits, particularly yield components (Dushyanthakumar, Shadadashari, 2007; Donoso-Ñanculao et al., 2015; Dewi, Dwimahyani, 2019; Rahayu, 2020).

Experimental trials designed to evaluate yield performance, stability, and adaptability are essential for characterizing genetic materials such as mutant lines before they are released as new breeding lines or varieties (Shu, 2012). Conversely, the observed variation in genotype grain yield across different environments highlights the need to carefully determine the yield performance and stability of genotypes in a range of environmental conditions (Ebadi et al., 2019). Therefore, it is crucial to investigate the yield stability of rice mutant lines and

to identify those lines that exhibit both high yield potential and stable performance across diverse environmental conditions. The likelihood ratio test (LRT) conducted in this study revealed significant effects of both the environment and genotype-byenvironment interaction (GEI). Similar results have been reported by other researchers conducting multi-environment trials with rice (Bose et al., 2011; Akter et al., 2015; Sharifi et al., 2017; Rahayu, 2020). Variations in environmental conditions from year to year and from location to location contribute to a pronounced GEI effect, which underscores the role of genetic factors in influencing genotype performance across different environmental conditions (Dia et al., 2016).

The restricted maximum likelihood (REML) analysis conducted in this study indicated that environmental variance contributed more significantly to the phenotypic variance observed for traits such as plant height, number of tillers, panicle length, number of filled grains per panicle, hundred-grain weight, and grain yield. In contrast, GEI had a greater contribution to the observed variance in the number of unfilled grains per panicle and fertility percentage. The first two principal components derived from the GEI analysis accounted for 68.13 and 14.46 % of the total genotype-by-environment interaction variation, respectively. In line with this finding, other researchers have acknowledged the substantial contribution of the first two principal components in explaining genotype-by-environment interaction effects on rice grain yield (Nayak et al., 2008; Akter et al., 2015; Allahgholipour, 2017; Rahayu, 2020).

Genotype-by-environment interaction reduces the predictability of genotype performance in target environments based on observations made in test environments (Yan et al., 2011). Numerous procedures and statistical methods have been developed to quantify genotype by environment interaction through the evaluation of genotypes across multiple environments. In addition to conventional multivariate methods like AMMI and GGE biplot analysis, the best linear unbiased predictor (BLUP) method has also been recommended for evaluating genotypes in different environments and for quantifying genotypic stability. Several researchers have employed the BLUP method to assess the stability of rice genotypes (Balestre et al., 2010; Donoso-Ñanculao et al., 2015). The use of restricted maximum likelihood (REML) for estimating variance components offers significant flexibility in analysis (Patterson, Thompson, 1971) and can effectively handle complex data structures (Searle et al., 1992).

The WAASBY index (Olivoto et al., 2019a) facilitates the weighting between mean performance and stability (MPE) in genotype evaluation. The primary advantage of this procedure is its integration of AMMI’s graphical tools (Gauch, 2013) with the predictive accuracy of BLUP for stability analysis (Piepho, 1994; van Eeuwijk et al., 2016). Consequently, the WAASB approach, which proposes the SVD (singular value decomposition) of a two-way table with BLUPs for GEI interaction (instead of the residual of the additive model as in standard AMMI), allows for a graphical representation of a random GEI structure. The WAASB index accounts for more than one (stability based on IPCA1) or two (AMMI-stability value) interaction principal component axes. In the present research, the first three principal components accounted for over 82 % of the GEI variation in grain yield, making the WAASB and WAASBY indices more effective in identifying superior genotypes. On the other hand, the variation of certain genotypes is explained by more IPCAs, making indices such as WAASB necessary, which is calculated using additional principal components. Consequently, we utilized this index and identified genotypes G1, G3, G5, G12, and G18 as superior (Fig. 2). The biplot based on this index was also employed to assess the discriminating ability of environments, revealing that the environments in the first two sections of biplot type III (Fig. 2), particularly E6, exhibited the highest WAASB value and strong genotype discrimination capability. However, the genotypes in these two quadrants with a significant contribution to the GEI were unstable. In the fourth quadrant of this biplot, genotypes G18, G1, G3, G5, and G12 emerged as the most desirable, demonstrating high yield stability and performance. The ranking of rice genotypes with varying weights for grain yield performance and stability in Figure 3 illustrated the diversity among rice mutant lines. In this heatmap, scenarios with different weights of yield stability and performance were simulated to display changes in genotype ranking. The grain yield of mutant lines G5, G12, G2, G7, and G1 surpassed that of parental varieties and Gilaneh, the control variety.

The HMRPGV method evaluates grain yield performance, stability, and adaptability simultaneously within a genotypic context (Resende, 2007). The HMRPGV of 1.306 for G5 indicated a 30.6 % increase in grain yield over the general mean. It appears that yield performance is prioritized over stability in the HMRPGV, and the selection of genotypes is based more on yield performance (as compared to Fig. 3) than on stability.

When data on several traits are available, the multi-trait stability index (MTSI) has many useful applications for simultaneous selection of mean performance and stability. This index is an MTSI-based on factor analysis, designed for simultaneous selection using both fixed and mixed effect models for multiple traits (Olivoto et al., 2019b). The factoranalytic variance-covariance structure accommodates different variances across environments and covariances between pairs of environments by approximating a completely unstructured covariance matrix through factor analysis. Therefore, it is reasonable to assume that the model will fit better than a standard mixed effect model. This factor analysis of WAASBY, which is the first step in computing the MTSI index, revealed that the eight traits were grouped into two factors. A Euclidean distance was then used to compute the distance between the genotypes’ scores and the ideotype’s score. This plot (Fig. 4) displays the genotype ranking in ascending order for the MTSI index. The selected genotypes using MTSI, including G7, G5, and G1, are shown in red, and the spiral depicts the cutpoint according to the selection pressure. A higher weight for mean performance was assigned to tiller number, panicle length, filled grains per panicle, fertility percentage, hundred grain weight, and grain yield. It is preferable to select highly productive genotypes that may not perform well in terms of broad stability and then consider the adaptability of these genotypes to specific environments to make better recommendations. The MTSI index enables simultaneous selection of genotypes based on performance and stability across all traits, making it a unique index (Olivoto et al., 2019a). This index has been used for the selection of superior genotypes in Solanum melongena (Koundinya et al., 2019), Brassica spp. (Bocianowski et al., 2019), chickpea (Sellami et al., 2021), maize (Olivoto et al., 2021), and rice (Sharifi et al., 2021).

MTSI serves as an effective tool for identifying the strengths and weaknesses of genotypes and selecting those with desired mean performance and stability (Olivoto et al., 2021). This approach enabled the identification of genotypes that combine the desired mean performance and stability (MPE) for important traits such as grain yield (GY), hundred-grain weight (HGW), fertility percentage (FP), and tiller number (TN) (Fig. 5).

## Conclusion

The significant ef﻿﻿fect of genotype indicated that the mutation process has successfully created a reasonable level of diversity in the genetic material. The first three principal components accounted for 92.35 % of the GEI variation with respect to grain yield, and the WAASB index was used to evaluate the stability of the genotypes. Biplot type III analysis revealed that genotypes G18, G1, G3, G5, and G12 were both high-yielding and stable across the tested environments. The heatmap visualization of yield performance and WAASB stability scores indicated that genotypes G3, G9, G6, G12, and G5 were highly productive and stable. The MTSI (multi-trait stability index) identified genotypes G7, G5, and G1 as the selected genotypes based on the overall stability and performance across all measured traits. According to the HMRPGV index, G5, G12, G7, G2, and G1 were identified as the top-performing genotypes. In conclusion, two genotypes, G12 and G5, exhibited a significant advantage over all other genotypes; however, only G5 was selected based on all traits in the MTSI index and could therefore undergo selection or cultivar introduction processes

## Conflict of interest

The authors declare no conflict of interest.
